# Automatic detection of major depressive disorder using electrodermal activity

**DOI:** 10.1038/s41598-018-35147-3

**Published:** 2018-11-19

**Authors:** Ah Young Kim, Eun Hye Jang, Seunghwan Kim, Kwan Woo Choi, Hong Jin Jeon, Han Young Yu, Sangwon Byun

**Affiliations:** 1 Bio-Medical IT Convergence Research Division, Electronics and Telecommunications Research Institute (ETRI), Daejeon, Korea; 2 Department of Psychiatry, Depression Center, Samsung Medical Center, Sungkyunkwan University School of Medicine, Seoul, Korea; 3 Department of Electronics Engineering, Incheon National University, Incheon, Korea

## Abstract

Major depressive disorder (MDD) is a common psychiatric disorder and the leading cause of disability worldwide. However, current methods used to diagnose depression mainly rely on clinical interviews and self-reported scales of depressive symptoms, which lack objectivity and efficiency. To address this challenge, we present a machine learning approach to screen for MDD using electrodermal activity (EDA). Participants included 30 patients with MDD and 37 healthy controls. Their EDA was measured during five experimental phases consisted of baseline, mental arithmetic task, recovery from the stress task, relaxation task, and recovery from the relaxation task, which elicited multiple alterations in autonomic activity. Selected EDA features were extracted from each phase, and differential EDA features between two distinct phases were evaluated. By using these features as input data and performing feature selection with SVM-RFE, 74% accuracy, 74% sensitivity, and 71% specificity could be achieved by our decision tree classifier. The most relevant features selected by SVM-RFE included differential EDA features and features from the stress and relaxation tasks. These findings suggest that automatic detection of depression based on EDA features is feasible and that monitoring changes in physiological signal when a subject is experiencing autonomic arousal and recovery may enhance discrimination power.

## Introduction

Major depressive disorder (MDD) is one of the most common psychiatric disorders, affecting more than 300 million people worldwide. According to recent estimates from the World Health Organization (WHO), depression is predicted to become the most common disease experienced by individuals of all ages, and the third largest contributor to disease burden by 2020^1^. Major depression is characterized by consistent irritability or feelings of sadness, and is associated with various symptoms, including sleep disturbances, loss of interest, persistent fatigue, reduced appetite, anxiety, and physical aches^2^. When severe, these symptoms can cause professional disability, which imposes a substantial economic burden on society owing to impaired work productivity^3^. Depression is also a risk factor for suicide, and, if left untreated, can lead to elevated mortality risk, and a serious public health concern^4^.

To diagnose a patient with MDD, psychiatrists use standard clinical criteria, such as those outlined in the Diagnostic and Statistical Manual of Mental Disorders (DSM)^5^. Although the DSM provides clear descriptions of symptoms and MDD diagnostic guidelines, diagnoses are often limited as they rely on patients’ subjective symptom reports, derived from clinical interviews and self-report questionnaires. As such, these do not provide an assessment of depression-related physiology or allow for an objective diagnosis^6^. Furthermore, the heterogeneous nature of depression makes it difficult to diagnose, with some reporting that even highly-trained clinicians are only able to agree on an MDD diagnosis between 4 and 15% of times^7–9^. Consequently, there has been great interest in developing more reliable methods to evaluate depression, which can significantly improve diagnostic accuracy and facilitate more precise treatment for MDD.

The use of physiological signals to assess autonomic nervous system (ANS) activity has attracted great interest in association with MDD, as accumulating evidence suggests that depression is related to ANS dysfunction. For example, previous research has shown that heart rate variability (HRV) was significantly altered in patients with MDD^10^. Similarly, electrodermal activity (EDA), which reflects sympathetic nervous system activity, is also sensitive to changes in clinical status^11^. Specifically, patients with depression exhibited lower skin conductance levels (SCLs) during rest than did healthy control subjects^12^. Similarly, stress-induced autonomic arousal, as measured by EDA, was found to be significantly reduced in MDD, indicating that depression may be associated with decreased autonomic responses to stimuli^13,14^. Also, EDA among MDD participants was found to be distinguishable from those with other psychopathologies, such as generalized anxiety disorder (GAD) or panic disorder (PD), as individuals with GAD and PD tended to exhibit autonomic hyper-activation^13,14^. These results suggest that autonomic activity, as represented by various physiological signals, serves as a quantitative marker of depression.

Based on these findings, recent studies on physiological measures proposed automatic detection of the depression using machine learning methods. For instance, HRV features, when combined with serum proteomics data, have been used to create support vector machine (SVM) algorithms for the diagnosis of MDD^15^. Valenza *et al*. used HRV parameters to assess depressive states in patients with bipolar disorder (BD) and also found that mood changes could be predicted using HRV nonlinear dynamics^16,17^. Ongoing work has focused on further developing data-driven strategies for the diagnosis of psychopathologies due to the recent success of machine learning in various medical and healthcare fields^18^.

While previous clinical research has suggested a potential role for EDA as a biomarker of MDD, only few have investigated how EDA data and machine learning methods can be used to objectively assess depression symptoms and render MDD diagnoses. The results in literature pertaining relevant EDA features in MDD has been inconsistent^19^. This poor consistency is likely a result of the heterogeneous presentation and multifactorial etiology of MDD, which substantially complicates research on this order^20^. Recently, Picard and colleagues predicted the severity of depressive symptoms in 12 patients with MDD by analyzing EDA, sleep patterns, motion, and other activities assessed via built-in smartphone sensors^21^. While this work is encouraging, the study was conducted without a healthy control comparison group and data were collected across multiple modalities to test prediction models. To the best of our knowledge, a more comprehensive clinical study, specifically focused on distinguishing patients with MDD from healthy controls solely using EDA data, has been rarely reported.

**Figure 1:**
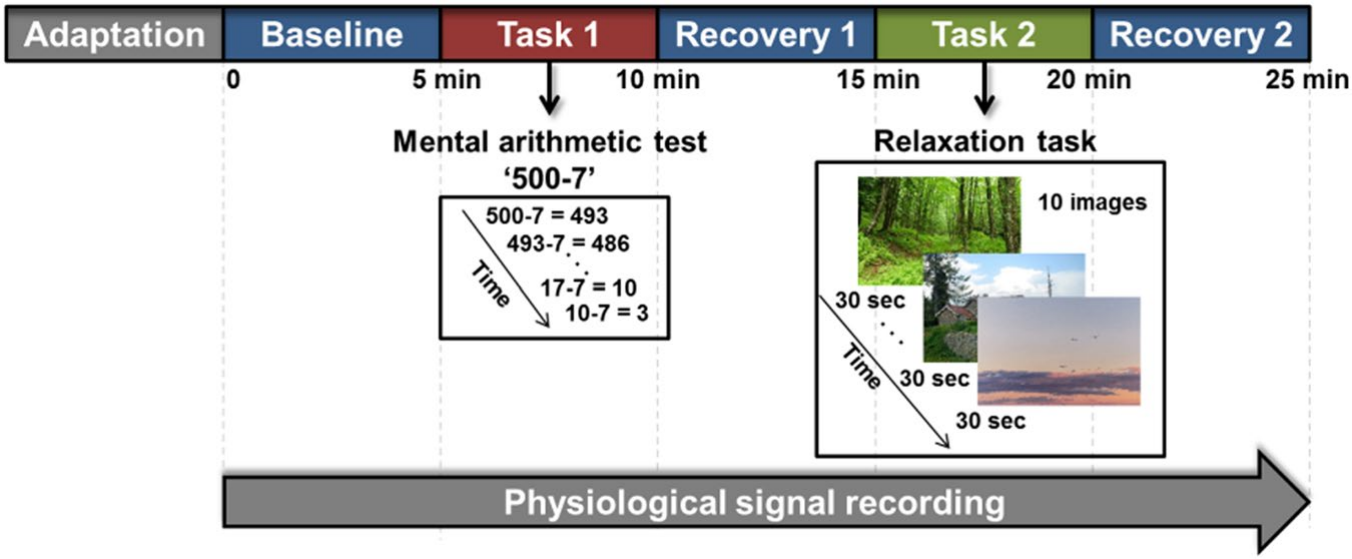
Experimental protocol. EDA signal was continuously recorded during five phases: baseline, mental stress task, recovery from the stress task, relaxation task, and recovery from the relaxation task. Mental stress in participants was induced by serial subtraction of 7 from 500. During the relaxation task, 10 images of natural scenery were presented to a subject. Each phase lasted for 5 min.

**Figure 2:**
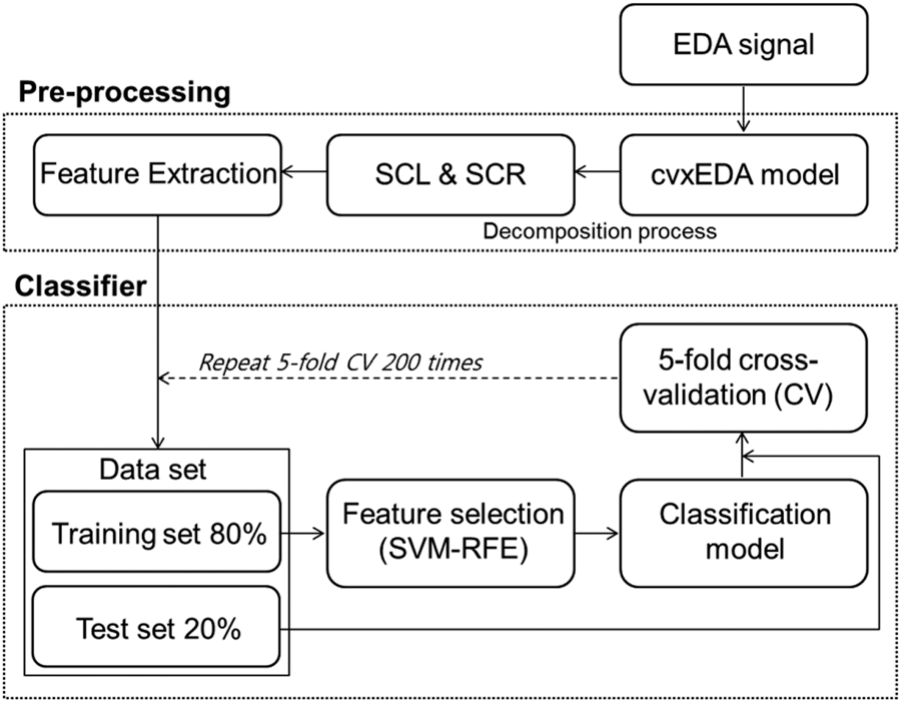
Overview of data processing.

**Figure 3:**
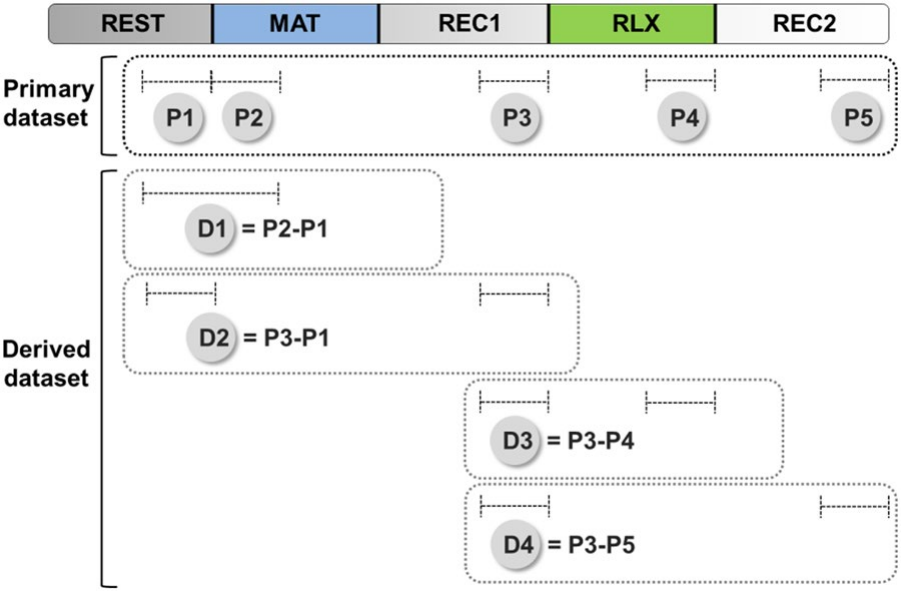
Schematic diagram describing primary and derived datasets. EDA features were assessed from a 150-sec period selected for each phase. The term “primary dataset” referred to the set of EDA features extracted from an individual phase (P1 to P5). The term “derived dataset” referred to the set of differential EDA features calculated from a pair of phases (D1 to D5). D1 represents the extent of reaction to mental stress. D2 estimates the difference in autonomic activity before and after the mental stress. D3 represents the extent of reaction to relaxation task. D4 estimates the difference in autonomic activity before and after the relaxation task.

Therefore, the objective of the present study was to investigate the feasibility of automatic differentiation of patients with depression from healthy controls using EDA and machine learning approaches. We measured EDA signals during five consecutive experimental phases: baseline, a mental arithmetic stress task, recovery from the stress task, a relaxation task, and recovery from the relaxation task (Fig. 1). Monitoring sympathetic activity and responses to various external stimuli may enable a better understanding of dysfunctional autonomic control in patients with MDD, possibly resulting in a more accurate diagnosis of their depression^22–25^. To represent autonomic activity, mean amplitude of SCL (MSCL), standard deviation of SCL (SDSCL), skewness of SCL (SKSCL), and non-specific skin conductance response (NSSCR) were extracted from each of the five phases (Supplementary Table S1). We also calculated differences in EDA features between two phases and used these parameters as input data to test their effectiveness in automatic discrimination (Figs 2 and 3). These multiple autonomic alterations may reveal abnormal autonomic control in patients with MDD^24,25^.

We have demonstrated that patients with MDD were differentiated from healthy control subjects with an accuracy of 74% using a decision tree classifier. Feature selection performed using a support vector machine recursive feature elimination (SVM-RFE) revealed that differential EDA features and features acquired from the stress and relaxation tasks were highly relevant. These findings suggest that automatic detection of depression based on EDA is feasible and that monitoring changes in physiological signal when a subject is experiencing autonomic arousal and recovery may enhance discrimination power.

## Results

### Descriptive statistics of subjects

Participants for the present study included 37 healthy controls (21 females) and 30 patients with MDD (22 females). Table 1 shows participants’ descriptive demographic and clinical characteristic statistics. There were no significant differences in sex, age, years of education, marital status, body mass index (BMI), or smoking status. Significant differences were detected in alcohol (*P*^*a*^ = 0.006) and caffeine use (*P*^*a*^ = 0.033) between the two groups. The MDD group had significantly higher Hamilton depression rating scores (HAM-D) (*P*^*a*^ < 0.001), Hamilton anxiety rating scores (HAM-A) (*P*^*a*^ < 0.001), and stress response inventory scores (SRI) (*P*^*a*^ < 0.001) than did the control group.

**Table 1:** Demographic and clinical characteristics of patients with MDD and healthy control subjects.

Factors	MDD (*N* = 30)	Control (*N* = 37)	*P*	*P* ^*a*^
Sex (%)			0.16 (*χ*^2^)	0.22
M	8 (27)	16 (43)		
F	22 (73)	21 (47)		
Age (SD)	42.5 (16.96)	41.3 (15.97)	0.95	0.95
Education, years (SD)	12.27 (4.21)	14.24 (2.58)	0.075	0.138
Marital status (%)			0.14 (*χ*^2^)	0.22
Single	13 (43.3)	16 (43)		
Married	13 (43.3)	21 (57)		
Divorced	2 (6.7)	0 (0)		
Bereavement	2 (6.7)	0 (0)		
BMI (SD)	22.61 (5.50)	22.37 (4.77)	0.75	0.825
Alcohol, frequency/week (SD)	0.32 (0.58)	0.83 (0.98)	0.002	0.006^*^
Smoking (%)			0.28 (*χ*^2^)	0.34
Nonsmoker	25 (83.3)	33 (89)		
Ex-smoker	2 (6.7)	0 (0)		
Current smoker	3 (10)	4 (11)		
Caffeine, cup/day (SD)	1.1 (1.89)	1.49 (1.2)	0.015	0.033^*^
HAM-D (SD)	16.23 (8.57)	2.29 (2.41)	<0.001	<0.001^*^
HAM-A (SD)	19.48 (6.95)	1.60 (1.56)	<0.001	<0.001^*^
SRI (SD)	17.87 (6.95)	1.57 (1.59)	<0.001	<0.001^*^

Two groups were compared by Mann-Whitney U test except for sex, marital status, and smoking, which were compared by chi-square test (*χ*^2^). *P*^*a*^ represents FDR adjusted *P-*value. Asterisks indicate statistically significant differences after the FDR adjustment (^*^P^*a*^ < 0.05).

Abbreviations: BMI, body mass index; HAM-D, Hamilton depression rating score; HAM-A, Hamilton anxiety rating score; SRI, Stress Response Inventory.

### Statistical analyses of EDA features

Participants were instructed to perform five different tasks while their EDA was measured (Fig. 1). The influence of both group and task on EDA features was statistically examined (Supplementary Table S2). We performed the non-parametric equivalent of a repeated-measures ANOVA as EDA features violated the normality assumption required for an ANOVA (for further details, see the Methods section)^26^. There were significant main effects of group and task on MSCL, SDSCL, and NSSCR. SKSCL was significantly affected by the task. No features revealed significant interactions between group and task.

The difference in an EDA feature between two experimental phases may be less affected by personal variation in ANS activity when compared to the same feature extracted from a single phase^27,28^. In the present study, four differential EDA features (dMSCL, dSDSCL, dSKSCL, and dNSSCR) were assessed from two distinct phases. We selected four pairs of phases, as shown in Fig. 3, to further account for various alterations in autonomic activity (for further details, see the Methods section). To avoid confusion, we use the term “primary dataset” to refer to the set of EDA features that were extracted from an individual phase (P1 to P5 in Fig. 3) and the term “derived dataset” to refer to the set of differential EDA data calculated from a pair of phases (D1 to D4 in Fig. 3). As a result, a total of 16 differential EDA features were assessed. When differential EDA features from the control and MDD groups were statistically compared, no significant differences were observed (Supplementary Table S3).

### Classification of control and MDD group participants based on EDA features

Four supervised machine learning algorithms were implemented to classify control and MDD participants based on their EDA features: SVM, decision tree, k-nearest neighbors (k-NN), and Naïve Bayes. Feature selection was performed using SVM-RFE, through which the most relevant features of a total of 36 (i.e., 20 features from primary datasets and 16 differential features from derived datasets) were identified. Performance measures were evaluated using 5-fold cross-validations repeated 200 times.

**Figure 4:**
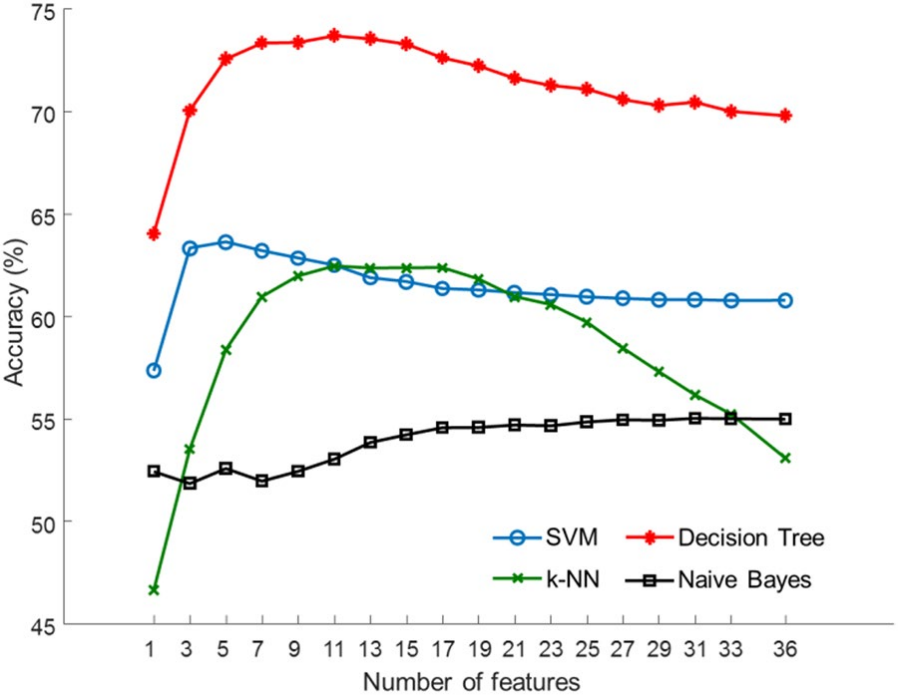
Average accuracy achieved with subsets of features selected by SVM-RFE.

**Figure 5:**
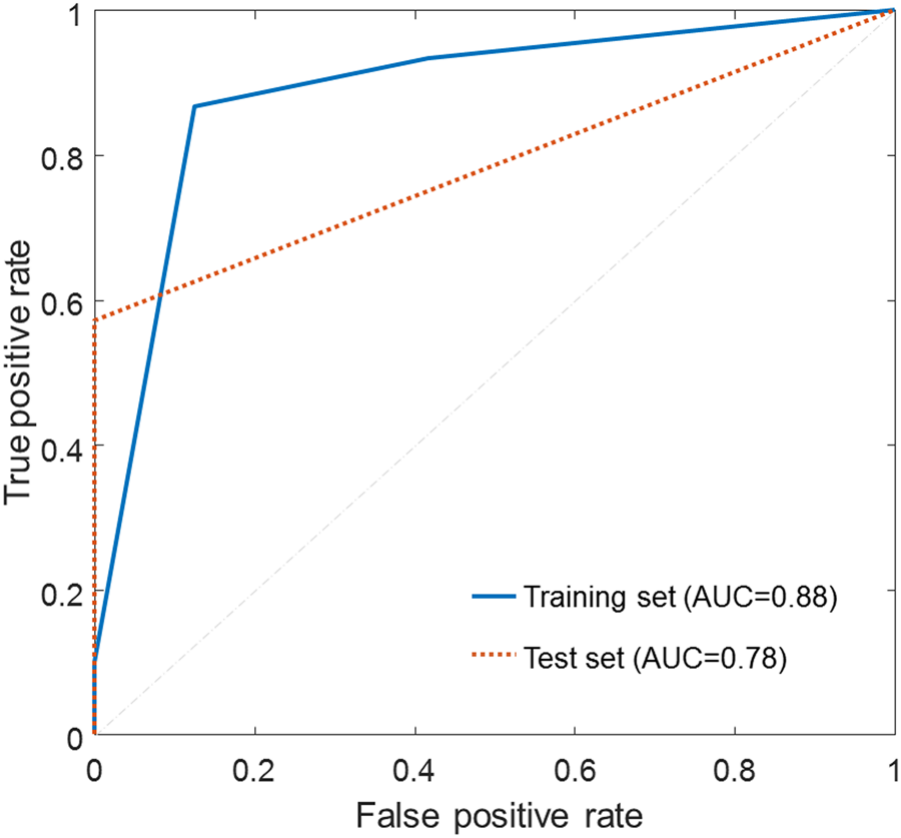
ROC curve analysis for the decision tree classifier during the use of the optimal features selected by SVM-RFE.

Figure 4 shows the classification accuracy as a function of the number of selected features. The decision tree outperformed SVM, k-NN, and Naïve Bayes in all tested subsets of features. Performance measures of the decision tree are summarized in Table 2. The best performance (73.71% accuracy, 73.74% sensitivity, and 71.15% specificity) was achieved using the 11 most relevant features. Supplementary Table S4 summarizes performance measures for SVM, k-NN, and Naïve Bayes classifiers. Receiver-operator characteristic (ROC) curves for the decision tree classifier during the use of optimal features are shown in Fig. 5, which depicts the comparison between results from the training and test sets. The areas under the curve (AUCs) for the training set and the test set were 0.88 and 0.78, respectively.

**Table 2:** Performance measures of the decision tree classifier assessed using 5-fold cross-validations repeated 200 times.

Classifier	Number of features	Accuracy (%)	Sensitivity (%)	Specificity (%)	PPV (%)	NPV (%)
Decision tree	1	64.03	66.03	54.53	65.32	72.17
	5	72.57	72.67	69.47	75.60	75.23
	**11**	**73**.**71**	**73**.**74**	**71**.**15**	**76**.**97**	**75**.**90**
	15	73.30	73.04	70.70	76.64	75.53
	20	72.00	71.94	68.87	74.99	74.69
	25	71.1	71.46	67.47	73.91	74.21
	30	70.38	70.85	66.00	72.97	74.13
	35	69.30	70.48	64.55	71.90	74.06

Feature selection was performed using SVM-RFE. The best performance is indicated in bold font.

Table 3 shows a list of the 36 EDA features sorted in descending order of average rank determined by SVM-RFE. The rank of each feature was averaged over 5-fold cross-validations repeated 200 times. The most relevant feature was MSCL from P1, followed by dMSCL from D2, dSKSCL from D3, SKSCL from P4 (relaxation task) and MSCL from P2 (stress task).

**Table 3:** Average ranks of the 36 EDA features determined by SVM-RFE.

Rank_Avg_	Dataset	Feature	Rank_Avg_	Dataset	Feature
1.4	P1	MSCL	19.8	P1	NSSCR
3.6	D2	dMSCL	20.0	D1	dNSSCR
4.8	D3	dSKSCL	20.8	P5	NSSCR
5.7	P4	SKSCL	21.7	P2	SDSCL
6.2	P2	MSCL	22.1	D1	dSDSCL
10.5	D2	dNSSCR	22.1	P3	SKSCL
11.3	D1	dMSCL	22.9	D4	dNSSCR
13.1	D3	dMSCL	23.4	P5	SKSCL
13.6	D4	dSDSCL	23.5	P2	NSSCR
15.9	P5	MSCL	23.9	P1	SKSCL
16.7	P4	NSSCR	24.9	D4	dSKSCL
17.1	D4	dMSCL	25.1	P1	SDSCL
17.2	P4	MSCL	25.4	D3	dNSSCR
17.6	P3	NSSCR	26.1	D2	dSKSCL
18.2	P5	SDSCL	26.7	P3	SDSCL
18.3	P3	MSCL	26.7	D3	dSDSCL
18.8	P2	SKSCL	30.7	P4	SDSCL
19.3	D1	dSKSCL	30.9	D2	dSDSCL

## Discussion

Our results suggest that EDA features measured during autonomic arousal and recovery may provide a promising biomarker for MDD. In the present study, we added a relaxation task to our experimental protocol, in addition to a stress task, to improve our classifier’s discrimination power. We also investigated the potential benefits of including differential EDA features in prediction. As summarized in Table 3, feature ranking performed using SVM-RFE revealed that differential features were highly informative. For example, the second and third most relevant features were differential EDA features, which were dMSCL from D2 and dSKSCL from D3, respectively. Six of the top 10 features were differential EDA features. In addition, the fourth and fifth-ranked features were measured during the relaxation task (SKSCL from P4) and stress task (MSCL from P2), respectively. These results suggest that EDA features, which account for various autonomic alterations, may play a major role in developing a predictive model of MDD.

The MDD group assessed here had significantly different MSCL, SDSCL, and NSSCR values from the control group (Supplementary Table S2). These results were consistent with previous studies, which have also reported significantly altered SCL and SCR in patients with MDD^19^. We also found a significant main effect of the task in MSCL, SDSCL, SKSCL, and NSSCR. In general, external stimuli elicit autonomic responses, causing physiological measures, such as SCL, to deviate from the basal activity^13^. Differential EDA features did not differ between the MDD and control groups although some differential EDAs were highly ranked by SVM-RFE. Since machine learning does not require prior assumptions about the relationships between variables, successful classification is not necessarily based on significant mean differences between the groups.

In the present study, we measured EDA to conduct machine learning-based detection of MDD. In previous studies that have used machine learning, HRV and electroencephalogram (EEG) have been widely studied as candidate physiological markers of psychiatric disorders and have demonstrated promising results. For example, HRV indices classified MDD and control groups with an accuracy of 88% using a linear discrimination analysis approach^25^. The prediction of mood states in BD patients achieved an accuracy of 60–83% using HRV parameters with an SVM classifier^17^. Additionally, biomarkers extracted from EEG and analysis performed using an artificial neural network distinguished between four patient classes (MDD, BD, schizophrenia, and normal controls) with an average accuracy of 85%^29^. A similar study involving EEG predicted antidepressant treatment effectiveness for patients with MDD with an accuracy of 88%^30^.

The results of the present study suggest that the relatively simple measurement of EDA can also provide clinically valuable information on the highly accurate assessment of MDD status in patients. Measuring EDA requires only two electrodes, which can be attached to minimally-obtrusive skin surfaces, such as the forearm^31^. Given this, our approach may be suitable for obtaining data while participants perform daily activities, as well as in more traditional clinical/experimental context. If a wearable device capable of real-time measurement of physiological signals is developed and integrated with prediction software, this technology could be implemented as a personalized healthcare system for real-time monitoring of patients. For example, a wrist-worn device with EDA and accelerometry sensors and machine learning capabilities has been successfully used to detect generalized tonic-clonic (GTC) seizure, demonstrating that wearable EDA sensors can indeed be used as a real-time disease tracking system^32^.

A limitation of the present study is that the classifications employed were based on a relatively small number of subjects (MDD = 30, control = 37). We are currently recruiting additional subjects and have been tracking longitudinal changes in EDA responses during a 3-month follow-up period. Further study of this cohort will allow us to extend our findings to develop a machine learning technique for disease diagnosis and severity assessment. We believe that these efforts will help us to build a more robust predictor of MDD for future use.

## Conclusion

We have demonstrated here, through proof-of-principle experiments, that EDA features can be used as a biomarker for MDD. Patients with MDD and healthy control participants were classified with 74% accuracy using a decision tree algorithm. The current study was specifically designed to test the feasibility of an EDA-based classification of patients with MDD. To increase discrimination power, EDA was measured while subjects underwent both stress-inducing and relaxation tasks. In addition to extracting EDA features from each phase, we also calculated differential features that represented differences in EDA between two distinct phases. Feature selection performed using SVM-RFE revealed that differential EDA features and features measured during the stress and relaxation tasks were highly useful for discrimination. Finally, these findings suggest that the machine learning method proposed and employed here, which accounts for multiple alterations in EDA, offers great potential as an objective marker of MDD which may ultimately improve patient diagnosis and treatment.

## Methods

### Subjects

This study was conducted between December 2015 and October 2016 at Samsung Medical Center, Seoul, South Korea. Participants included 30 patients with MDD and 37 healthy controls. Senior psychiatrists diagnosed all patients using DSM-IV MDD criteria. On screening, those patients who scored greater than or equal to 16 on HAM-D were recruited into the MDD group^33^. Healthy participants without any medical history of psychiatric disorders who responded to our advertisements were recruited into the control group. All subjects were informed about the purpose and method used in the present experiment and signed a written informed consent form. They were also financially compensated ($50) for their participation. This study was approved by the Institutional Review Board of Samsung Medical Center of Seoul, Korea (No. 2015-07-151) and performed according to all relevant guidelines.

### Procedure

The experimental protocol used in the current study was design to assess autonomic responses to stress and relaxation tasks (Figs 1 and 3). The whole procedure consisted of five phases, each of which lasted 5 min. We recorded physiological signals, including EDA, while subjects performed a specific task in each experimental phase. The first phase (‘REST’) consisted of a rest period during which subjects were instructed to sit comfortably while minimizing any movement. The second phase (‘MAT’) used a mental arithmetic test to induce mental stress in participants^34,35^. Here, we asked subjects to subtract serial 7′s, starting from 500, and verbally report their answers. If a mistake was made, the experimenter told the subject to repeat the calculation. In the third phase (‘REC1’), subjects were instructed to stop the arithmetic calculations and rest. During this phase, autonomic recovery from the stress task was assessed. During the fourth phase (‘RLX’), subjects were asked to relax while watching 10 consecutive images presented on a PC monitor. Each image depicted natural scenery and lasted for 30 sec. In the fifth and final phase (‘REC2’), image presentation ceased, and subjects were instructed to rest. During this phase, recovery from relaxation was assessed.

### Physiological recordings

Before measurement began, subjects were seated in a comfortable armchair with a headrest. The experimenter then explained the measurement procedure to the subject. Electrodes used to sense physiological signals were attached. Subjects were then allowed to acclimate to the laboratory environment. We used ProComp Infiniti (SA7500, Computerized Biofeedback system, Thought Technology, Canada) to record EDA, as well as patient electrocardiogram (ECG), photoplethysmogram (PPG), respiration, skin temperature, and electroencephalogram (2-channel EEG for Fp1, Fp2) measures. While in the present study, we only analyzed EDA values, these other measures were taken as part of a larger study. EDA was continuously recorded using SC-Flex/Pro sensors (SA 9308 M, Thought Technology, Canada). A constant electrical voltage (0.5 V) was applied between two dry Ag/AgCl electrodes, which were strapped to the distal phalanges of index and ring fingers of the subject’s non-dominant hand. The sampling rate was 256 Hz. Measurements were made in a humidity- and temperature-controlled room (23 °C and humidity below 50%).

### Pre-processing and feature extraction

All EDA data were processed in MATLAB (Mathworks, MA, USA). Figure 2 depicts an overview of our data processing pipeline. After removing motion artifacts, EDA signals were filtered using a second-order Butterworth low-pass filter (1 Hz cutoff, IIR) and a moving average filter to reduce signal noise. Then, the EDA signals were decomposed using a convex optimization approach (cvxEDA)^36^. The cvxEDA model describes EDA as the sum of three components, tonic component, phasic component, and an additive white Gaussian noise. The cvxEDA algorithm is available online from Mathworks File Exchange (www.mathworks.com/matlabcentral/fileexchange/53326-cvxeda). In the present study, fixed values of α = 0.008, *τ*_1_ = 0.7s, *τ*_2_ = 2s and γ = 0.01 were used for cvxEDA parameters. The tonic component, represented by skin conductance level (SCL), reflects the overall degree of arousal, which increases with alertness and decreases with relaxation^37^. The phasic component, represented by skin conductance response (SCR), reflects the short-time responses to external stimuli (1–5 sec after stimulus onset)^38,39^. From these, we calculated four features, mean amplitude of SCL (MSCL), standard deviation of SCL (SDSCL), skewness of SCL (SKSCL), and non-specific SCR (NSSCR), as summarized in Supplementary Table S1^38^.

As explained above, the experimental protocol was composed of five phases (REST, MAT, REC1, RLX, REC2). A 150-sec interval was selected for each phase, and EDA features during this interval were calculated (Fig. 3). We selected specific time windows to ensure that EDA features accurately reflected activated or recovered levels of autonomic activity in each phase. The length of the window was 150 sec, which was 25% of the total duration of the phase, which was 600 sec (5 min) long. A similar approach was used in previous studies. For example, Brouwer *et al*. used 2-min long stimuli to induce emotional states and evaluated skin conductance during the first 30-sec period, which was 25% of the total length of the stimulus interval^40^. Pruneti *et al*. used three experimental phases consisting 6-min rest, 4-min stress, and 6-min recovery phases. They used the last minute of the rest and recovery phases each (1/6 = 16.7%) and the first minute of the stress phase (1/4 = 25%) to evaluate SCL. In our study, we chose a conservative ratio, i.e., 25%, to define the window length^14^. In the present study, the final 150-sec period was selected to represent baseline activity for the REST phase. For the MAT phase, the first 150-sec period was used to measure activation before habituation that could affect measured activation levels. For the REC1 and REC2 phases, the final 150-sec was used to allow sufficient recovery time. Similarly, we selected the final 150-sec of the RLX phase to provide participants with enough time to respond to the relaxation task. A set of EDA features obtained from this individual phase is referred by the term “primary dataset” as shown in Fig. 3.

Also, four differential EDA features (dMSCL, dSDSCL, dSKSCL, and dNSSCR) were calculated from two distinct phases. In the present study, we selected four pairs of phases and used the term “derived dataset” to refer to a set of differential EDA data calculated from a pair of phases (Fig. 3). To measure the extent of reaction to the mental stress phase, EDA features from the REST phase were subtracted from those from the MAT phase (D1). To estimate the difference in autonomic activity from before and after stress, EDA features from the REST phase were subtracted from those from the REC1 phase (D2). Similarly, reactivity to the relaxation task was measured by subtracting EDA features from the RLX phase from those from the REC1 phase (D3). To estimate the difference in autonomic activity before and after the relaxation task, EDA features from the REC2 phase were subtracted from those from the REC1 phase (D2). A total of 16 differential EDA features were calculated.

### Statistical analyses

Statistical analyses were performed using MATLAB and R software (The R Foundation for Statistical Computing, Vienna, Austria). Mann-Whitney U tests were used to compare age, years of education, BMI, alcohol use, caffeine use, HAM-D, HAM-A, and SRI between the MDD and control groups, as these factors were not normally distributed. Sex, marital status, and smoking between the two groups were compared by chi-square tests. All four EDA features violated the normality assumption required for an ANOVA. Therefore, the effects of group and task on EDA features were tested by the non-parametric equivalent of a repeated-measures ANOVA using the R statistics package “nparLD”^26^. This method was developed to assess longitudinal data from repeated measurements based on a factorial design. The non-parametric test provided ANOVA-type statistics for examination of the following hypotheses: no between-subjects effect, no within-subject effect, and no interaction. In the present study, group was used as the between-subjects factor and task as the within-subject factor. Further methodological details can be found in the previous study conducted by Brunner and Puri^41^. Mann-Whitney U tests were used to compare differential EDA features between the MDD and control groups, as these features did not meet the normality assumptions. To control type I error of multiple comparisons, *P*-values were adjusted for the false discovery rate (FDR) using the Benjamini-Hochberg method at a level of 0.05^42^. For all statistical tests, an adjusted *P*^*a*^-value of less than or equal to 0.05 was considered significant.

### Feature selection and classification

We used four supervised machine learning algorithms, support vector machine (SVM), decision tree, k-nearest neighbors (k-NN), and Naïve Bayes, to classify the MDD and control groups using EDA features as input data. As shown in Fig. 2, the 5-fold cross-validation method was used to split data and evaluate classifier performance. Training was performed using 80% of the total data set (4 of 5 folds) and testing was performed with the remaining 20%. We employed an SVM-RFE algorithm as a feature selection method; this algorithm ranked features based on a backward sequential selection method, which removed the features one by one^43^. To ensure that all classifiers were trained using the same training data and the same subset of features, SVM-RFE was used to first determine the feature rankings for a given training data set. Then, the ranking results and the same training set were applied to train SVM, decision tree, k-NN, and Naïve Bayes models. The prediction error of each classifier model was evaluated using the unseen test data set. This process was repeated five times for each fold to complete the 5-fold cross-validation. It is important to note that cross-validation was external to the feature selection process to evaluate the prediction error accurately. To reduce variance in the prediction error, the 5-fold cross-validation was repeated 200 times, and validation results from all repetitions were averaged to finally report the model’s predictive performance^44^. We also averaged the rank of each feature from the 200 repeats to provide an estimate of the relative importance of the feature.

In binary classification, SVM maps training data into a multidimensional feature space and finds a hyper-plane in the feature space that maximizes the distances between two classes^45^. For both SVM-RFE and classification, we used a linear kernel function with the regularization parameter *C* = 1. Decision tree performs classification using a recursive binary partition of target variables^46,47^. A decision tree model begins at the root node and branches to internal nodes. Decisions to split are made by impurity measures, such as the Gini index or information gain, and this process continues until a final node is reached with a predicted class value. Here, we used the Gini index for impurity measures. A k-NN model is a type of instance-based learning^48^. A k-NN classifier estimates the class label of a new observation by the majority class of the k closest neighbors according to a distance metric. In the present study, the k value was set to three, and Euclidean distance was used as the distance metric. Naïve Bayes is a probabilistic learning method based on the application of Bayes’ theorem^49^. It estimates the parameters for a feature’s probability distribution using training data and assuming that predictors are conditionally independent in every class. Then, with new test data, Naïve Bayes computes the posterior probability of that new data belongs to each class and chooses the class with the largest posterior probability. For the present study, we assumed that EDA features followed a Gaussian distribution. We adopted accuracy, sensitivity, specificity, positive predictive value (PPV), negative predictive value (NPV), and area under the curve (AUC) as the performance measures.

## Electronic supplementary material

Supplementary information
