# Supplementary material for: Automatic detection of major depressive disorder using electrodermal activity
Source: Sci Rep. 2018 Nov 19;8:17030. doi: 10.1038/s41598-018-35147-3 (PMC6242826; doi:10.1038/s41598-018-35147-3)
Supplement: Supplementary file 1 — Supplementary information [file 41598_2018_35147_MOESM1_ESM.pdf]

Supplementary Information

Automatic detection of major depressive disorder using electrodermal activity

Ah Young Kim[1], Eun Hye Jang[1], Seung-Hwan Kim[1], Kwan Woo Choi[2], Hong Jin Jeon[2], Han Young Yu[1,*], Sangwon Byun[3,*]

[1]Bio-Medical IT Convergence Research Division, Electronics and Telecommunications Research Institute (ETRI), Daejeon, Korea

[2]Department of Psychiatry, Depression Center, Samsung Medical Center, Sungkyunkwan University School of Medicine, Seoul, Korea

[3]Department of Electronics Engineering, Incheon National University, Incheon, Korea

[*]Corresponding authors

Correspondence and requests for materials should be addressed to H.Y.Y.
(email: uhan0@etri.re.kr) or S.B (email: swbyun@inu.ac.kr)

Feature	Definition	Formula
MSCL	Mean amplitude of SCL	$\frac{1}{N}\sum_{n=1}^{N} X_n$, where $X_n = \mathrm{SCL}$
SDSCL	Standard deviation of SCL	$\left(\frac{1}{N-1}\sum_{n=1}^{N}(X_n - \mu_X)^2\right)^{\frac{1}{2}}$, where $\mu_X = \mathrm{MSCL}$
SKSCL	Skewness of SCL	$\frac{G_3}{G_2\sqrt{G_2}}$, where $G_k = \frac{1}{N}\sum_{n=1}^{N}(X_n - \mu_X)^k$
NSSCR	Non-specific SCR	$\frac{1}{N}\sum_{n=1}^{N} Y_n$, where $Y_n = \mathrm{Amplitude\ of\ SCR}$

Supplementary Table S1. Description of EDA features.

Feature	Group effect (MDD vs. control)			Task effect (5 tasks)			Interaction (group × task)		
	F	*P*	P^a	*F*	*P*	P^a	*F*	*P*	P^a
MSCL	11.60	< 0.001	0.002*	45.92	< 0.001	< 0.001*	0.60	0.610	0.732
SDSCL	6.21	0.013	0.025*	53.90	< 0.001	< 0.001*	0.09	0.973	0.990
SKSCL	0.94	0.333	0.455	31.46	< 0.001	< 0.001*	1.13	0.341	0.455
NSSCR	5.11	0.024	0.041*	30.02	< 0.001	< 0.001*	0.06	0.990	0.990

Supplementary Table S2. Effects of group and task on EDA. Statistical analyses were performed with the non-parametric equivalent of a repeated-measures ANOVA using the R statistics package “nparLD”. Group was used as the between-subjects factor and task as the within-subject factor. P^a represents FDR adjusted *P*-value. Asterisks indicate statistically significant effects after the FDR adjustment ($^*P^a < 0.05$).

Derived dataset	Differential feature	MDD	Control	P	P^a
		Mean (SD)	Mean (SD)		
D1	dMSCL	0.52 (0.51)	0.87 (1.02)	0.119	0.325
	dSDSCL	0.05 (0.09)	0.19 (0.63)	0.054	0.325
	dSKSCL	-1.40 (1.69)	-0.95 (1.07)	0.468	0.687
	dNSSCR	0.06(0.10)	0.24 (0.54)	0.073	0.325
D2	dMSCL	0.43 (0.57)	0.58 (1.35)	0.243	0.432
	dSDSCL	0.01 (0.04)	0.01 (0.33)	0.142	0.325
	dSKSCL	-0.13 (1.33)	0.26 (1.26)	0.524	0.687
	dNSSCR	0.01 (0.06)	0.01 (0.26)	0.519	0.687
D3	dMSCL	0.16 (0.32)	0.26 (0.63)	0.122	0.325
	dSDSCL	0.01 (0.03)	-0.09 (0.58)	0.796	0.849
	dSKSCL	-0.18 (0.67)	0.17 (0.80)	0.059	0.325
	dNSSCR	0.01 (0.06)	-0.09 (0.37)	0.142	0.325
D4	dMSCL	0.13 (0.50)	0.36(1.15)	0.210	0.420
	dSDSCL	0.01 (0.04)	0.04 (0.31)	0.558	0.687
	dSKSCL	-0.08 (1.21)	0.34 (1.59)	0.618	0.706
	dNSSCR	-0.01 (0.06)	-0.05 (0.13)	0.934	0.934

Supplementary Table S3. Group comparison of differential EDA features (Mann-Whitney test between the MDD and control groups, P^a represents FDR adjusted P-value).

Classifier	Number of features	Accuracy (%)	Sensitivity (%)	Specificity (%)	PPV (%)	NPV (%)
SVM	1	57.35	54.05	61.65	65.56	53.67
	5	63.62	63.69	54.65	65.56	71.30
	11	62.49	61.53	55.07	64.94	68.85
	15	61.69	60.90	53.80	64.04	68.46
	20	61.24	60.42	52.88	63.54	68.40
	25	60.95	60.05	52.58	63.28	68.13
	30	60.81	59.87	52.37	63.15	68.04
	35	60.79	59.86	52.30	63.12	68.07
k-NN	1	46.64	46.34	88.02	54.20	46.00
	5	58.37	56.70	49.82	60.91	65.70
	11	62.44	62.24	49.98	63.56	61.30
	15	62.35	63.73	47.83	63.19	61.32
	20	61.33	62.12	46.53	62.17	60.27
	25	59.71	59.89	45.03	60.82	58.66
	30	56.71	54.92	42.33	58.50	55.68
	35	53.72	49.63	40.62	56.37	52.79
Naïve Bayes	1	57.35	48.86	76.98	63.04	31.39
	5	63.62	49.36	84.20	63.23	25.49
	11	62.49	49.34	83.95	64.36	25.83
	15	61.69	50.36	84.72	68.12	28.09
	20	61.24	50.69	84.93	69.42	28.77
	25	60.95	50.81	84.92	69.62	29.06
	30	60.81	50.93	85.55	69.63	28.76
	35	60.79	50.93	85.57	69.55	28.80

Supplementary Table S4. Performance measures of support vector machine (SVM), k-nearest neighbors (k-NN), Naïve Bayes models estimated using 5-fold cross-validations repeated 200 times.
